# The use and subjective experience of sleep apps and their relationship with personality characteristics among young adults

**DOI:** 10.3389/frsle.2025.1499802

**Published:** 2025-02-05

**Authors:** Ståle Pallesen, Øystein Vedaa, Erlend Sunde, Anette Harris, Siri Waage, Ingvild West Saxvig, Jiewen Zhang, Bjørn Bjorvatn

**Affiliations:** ^1^Department of Psychosocial Science, University of Bergen, Bergen, Norway; ^2^Norwegian Competence Center for Sleep Disorders, Haukeland University Hospital, Bergen, Norway; ^3^Department of Health Promotion, Norwegian Institute of Public Health, Bergen, Norway; ^4^Department of Sociology, Central South University, Changsha, China; ^5^Department of Global Public Health and Primary Care, University of Bergen, Norway

**Keywords:** sleep, app, effects, users, survey

## Abstract

**Introduction:**

Sleep app use has become widespread in recent years. Still, understanding of characteristics of users and the impact of sleep app usage remains limited.

**Methods:**

A sample of 940 young adults (18–30 years) living in the UK were recruited from Prolific (online research platform offering researchers access to survey panels) and invited to participate in a survey on the use and experience with sleep apps. Both sleep app users and non-users were invited. The survey included questions about demographics, morningness (Horne–Östberg Morningness–Eveningness Questionnaire-reduced scale), insomnia symptoms (Bergen Insomnia Scale), personality (HEXACO: Honesty-Humility, Emotionality, Extraversion, Agreeableness, Conscientiousness, and Openness to Experience), in addition to sleep app usage and how such use is experienced. A logistic regression analysis was conducted to identify characteristics of sleep app users. Factor analysis was used to elucidate dimensions of sleep app use experiences. Scores on the items making up each factor were then regressed on demographic variables, morningness, insomnia symptoms and personality.

**Results:**

In all, 36.8% of the participants reported using or having used sleep apps. Use was positively associated with age, insomnia symptoms, and conscientiousness. The factor analysis revealed three factors coined: trust/objectification (trust in sleep app results), negative (e.g., becoming stressed) and positive (e.g., prioritize sleep more) perceived effects. Trust/objectification was positively associated with age and agreeableness, and inversely related to high education and openness to experience. Insomnia symptoms and emotionality were associated with perceived negative effects. Morningness and male gender were associated with perceived positive effects whereas high education had an inverse relationship with perceived positive effects.

**Discussion:**

The use of sleep apps was widespread. Different responses to sleep app usage were observed and linked to various individual variables. The findings suggest that perceived effects of sleep apps usage should be considered in light of individual differences. Research on sleep apps is still in its early stages, and several suggestions (recommendations) for future studies are outlined.

## 1 Introduction

During recent years sleep and sleep related issues have become increasingly popularized in terms of bestselling books (Pelayo, 2020; Walker, 2017), YouTube videos (Monten et al., 2022), and internet (e.g., www.sovno.no) information pages (Lee et al., 2018; Turan and Erdogan, 2018). The focus on sleep has not been missed out by commercial interests, and the term “sleep industry” has accordingly been used to reflect efforts taken by various commercial interests to offer everything from nightwear, pharmacological sleep aids, and the ultimate bed (Coveney et al., 2023). A novel type of product manufactured by the sleep industry is the numerous sleep apps which have become readily available. Many sleep apps are multipurpose, typically addressing activity and health, whereof sleep entails one aspect of this (Shelgikar et al., 2016). In line with the latter notion, sleep apps seem to be part of a wider trend denoted as the “quantified self-movement” or “self-tracking”, implying that people use new technology to count and monitor various phenomena such as nutritional intake, the number of steps walked, energy expenditure, heart health, among others (Lupton, 2013). Sleep apps typically monitor sleep (or more precisely activity levels, pulse, temperature or other type of data), and may allegedly also be used to assess sleep problems and disorders, as well as suggest ways to improve sleep, although it is emphasized that consumer sleep technology should not replace validated diagnostic instruments (Khosla et al., 2018). The vast majority of sleep apps are based on sensors (e.g., for sound and movement) built into mobile phones (Choi et al., 2018). Others rely on wearable sensors (e.g., microphones, accelerometers, temperature sensors, heart rate monitors, SpO_2_ sensors, ECG sensors, and sensors for peripheral arterial tone) placed on the body, wrist, finger or embedded in clothing or in the sleep environment (Ko et al., 2015).

A distinction is normally drawn between passive vs. active app functions. Passive sleep app functions typically entail sleep tracking, audio environment monitoring, including recordings of snoring and sleep talking, and the estimation of various sleep parameters, such as total sleep time and sleep efficiency, including the detection of possible sleep problems. Active sleep app functions reflect efforts to impact sleep such as audio feedback to promote sleep and dreaming, manage ideal wake-up times, and facilitate relaxation and allegedly even treat insomnia (Al Mahmud et al., 2022).

Both positive and negative effects of sleep apps on consumer's sleep and health have been noted. Possible positive effects include increased involvement and self-care, motivating healthy behaviors, and becoming involved in research, hence sleep apps could be a valuable tool to help people achieve better sleep (Van den Bulck, 2015). In some instances, sleep apps may convey clinical information about sleep problems, which may help the person being taken more serious by their doctor, which in turn may facilitate referral to proper treatment or follow-up assessment. On the negative side, sleep apps show poor correspondence with subjective sleep experiences and the gold standard for sleep measurement, polysomnography (Al Mahmud et al., 2022; Bhat et al., 2015; Fino et al., 2020). Many sleep apps include various recommendations and sleep promoting propositions, however a literature review documented that few of these had a firm empirical and clinical basis (Lee-Tobin et al., 2017). Furthermore, sleep apps may be used for self-diagnosing of sleep disorders, risking a large number of false positives and which may put unnecessary toll on health care services. In addition, some individuals may develop an obsession with healthy sleeping, which has been denoted orthosomnia, due to feedback from sleep apps (Baron et al., 2017; Van den Bulck, 2015). Some have also expressed concerns related to potential data privacy and potential misuse of data collected through sleep apps (Ananth, 2021).

Few studies have investigated characteristics of sleep app users and their experiences with using such apps. In one study, two reviewers and 30 users evaluated six mobile apps for sleep tracking. Between 30% and 40% of the users agreed that the app increased awareness of sleep, knowledge about sleep hygiene, attitudes toward improving sleep hygiene, intention to change, help seeking behavior and sleep related behavioral change (Karasneh et al., 2022). In another study, user reviews of sleep apps by Chinese and US users were analyzed, showing satisfaction rates of 56.6% and 45.9%, respectively. User satisfaction was mostly related to the app's sleep promotion effects and sleep advice functions, whereas user dissatisfaction was primarily related to the app's stability, compatibility, value of money, and sleep tracking function (Nuo et al., 2023). A longitudinal study showed that perceived usefulness was positively related to usage and well being, which align with technology acceptance theories (Attie and Meyer-Waarden, 2023).

Nevertheless, there is limited knowledge about the number of people using sleep apps, individual factors that are associated with the use of and experiences of sleep app users. In addition to demographics, sleep app use and experiences thereof may be related to the morningness–eveningness dimension. Overall, evening types seem to have poorer sleep quality than morning types and also use computer and other modern digital devices more often than morning types (Minz and Pati, 2021). Due to the latter, a negative association between sleep app use and morningness may be expected. Another potential sleep app use related variable is insomnia, which denotes problems initiating or maintaining sleep and results in some form of daytime impairment (American Academy of Sleep Medicine, 2014). Insomnia is the most common sleep disorder, and is often comorbid with other sleep disorders as well as with psychological or somatic disorders and diseases (Bjorvatn et al., 2021). People with insomnia have, for example, been characterized by hyperarousal (Dressle and Riemann, 2023) and worry (Jansson and Linton, 2006), and interestingly, also by the use of various sleep-inducing techniques (Bjorvatn et al., 2023). Hence, a positive association between insomnia and sleep app use seems conceivable.

In terms of personality the five-factor model is one of the most recognized trait models today (Digman, 2002; Wiggings, 1996) and posits that personality consists of five relatively stable and unrelated personality dimensions: Neuroticism/emotionality (reflecting dysphoric states such as nervous tension, depression, frustration, guilt, and self-consciousness), conscientiousness (being thorough, neat, well-organized, diligent, achievement-oriented), agreeableness (involving altruism and trust, empathy, and nurturance), openness to experience (associated with creativity, intellectual interests, differentiated emotions, aesthetic sensitivity, need for variety, and unconventional values), and extroversion (linked to being cheerful, enthusiastic, optimistic, energetic, talkative, sociable, and warm) (Digman, 2002; Wiggings, 1996). Overall, results seem to suggest quite consistently that conscientiousness and neuroticism/emotionality are positively and negatively associated with sleep quality, respectively (Dekker et al., 2017; Duggan et al., 2014; Krizan and Hisler, 2019). More recently, a six dimensional personality model, the HEXACO, has been developed. The model shares fundamental similarities with the five factor model of personality, but includes an additional sixth dimension, honesty- humility, which encompasses fairness, sincerity, greed-avoidance, and modesty (Lee and Ashton, 2004). However, there is limited knowledge regarding the relationship between this personality dimension and sleep related behaviors. Against this backdrop we conducted a study investigating how demographic factors, morningness, insomnia symptoms, and the HEXACO-personality traits relate to both the usage of sleep apps and the experiences associated with their use.

## 2 Materials and methods

### 2.1 Sample and procedure

Respondents between the ages of 18 and 30 were recruited via Prolific (https://www.prolific.co), a UK based company that recruits participants for paid surveys. The survey focused on young adults as studies have shown this group is more likely to use health apps than their older counterparts (Naszay et al., 2018). Data collection took place in May 2023. Participants were located in the UK and had at least 95% approval rate from other research projects and at least 10 previous study participations on the Prolific platform. They were invited to take part in an online survey comprising questions related to demographics, morningness–eveningness, insomnia symptoms, personality traits in line with the HEXACO-model, use of sleep apps, and experienced effects of such apps. Each participant was compensated with £5 for completing the survey. All participants provided informed consent before participating. A total of 1,037 participants were recruited, of whom 982 completed the survey. Since online surveys may be susceptible to careless responses, such as inattentive and random response styles (Meade and Craig, 2012), non-valid responses may influence the findings. In order to ensure data quality, three questions to identify careless responding in line with Brühlmann et al. (2020) were adjusted to fit seamlessly with the layout and formulation of the questionnaire: “Respond with strongly agree for this item”, “Respond with definitely agree for this item”, and “The shape of the Earth is…” (flat/round). Data from 12, 19, and 11 respondents, respectively, were not included as they failed these attention checks. Consequently, the analytic sample comprised 940 participants (476 females, 451 males, 13 others; *M*_age_ = 25.51, SD = 3.28). A total of 513 were single/divorced/separated whereas 427 were married or had a partner. The vast majority categorized themselves as white/Caucasian (*n* = 759), whereas a minority were mixed (*n* = 40), Asian (*n* = 98), Black/African/Caribbean (*n* = 27), Arab (*n* = 3) or belonged to any other ethnic group (*n* = 13).

### 2.2 Instruments/questionnaires

#### 2.2.1 Demographics

Questions about age and gender were included. The respondents were also asked if they had any child caretaker responsibilities, ranging from 0 up to 6 children or more. This variable was dichotomized into no/yes. In terms of education the respondents were grouped into three categories: no formal qualification/secondary education/high school (*n* = 286), technical or community college/undergraduate degree (*n* = 472), and graduate or doctorate degree (*n* = 181). Questions about marital status and ethnicity were also included.

#### 2.2.2 Morningness

The Horne–Östberg Morningness–Eveningness Questionnaire-reduced scale (rMEQ) (Adan and Almirall, 1991) was used to measure the eveningness-morningness dimension. It comprises five items, and higher composite scores indicate higher levels of morningness. The Cronbach's alpha of the rMEQ was 0.72 in the current study.

#### 2.2.3 Insomnia symptoms

The Bergen Insomnia Scale (BIS) was used to measure insomnia symptoms (Pallesen et al., 2008). It was constructed based on the inclusion criteria for insomnia symptoms in the Diagnostic and Statistics Manual for Mental Disorders, 4th edition (DMS-IV) (American Psychiatric Association, 1994). The BIS comprises six items that refer to difficulties with sleep onset, sleep maintenance, early morning awakening, non-restorative sleep, daytime impairment, and dissatisfaction with sleep. Each item is scored from 0 to 7 reflecting the number of days per week the symptom in questions is experienced. Higher total scores indicate higher levels of insomnia symptoms (Pallesen et al., 2008). Cronbach's alpha was 0.83 in the current study.

#### 2.2.4 Personality

The HEXACO-60 was administered to assess personality traits and provides composite scores of six dimensions, each reflected by 10 items: Honesty-Humility (H), Emotionality (E), Extraversion (X), Agreeableness (A), Conscientiousness (C), and Openness to Experience (O). Participants responded to each item on a scale from 1 (strongly disagree) to 5 (strongly agree). Higher total scores for each dimension indicate higher levels of the respective trait (Ashton and Lee, 2009). Cronbach's alpha for the dimensions: H, E, X, A, C, and O was 0.72, 0.81, 0.84, 0.78, 0.78, and 0.80, respectively.

#### 2.2.5 Sleep app use and sleep app use experience

A question about current or previous sleep app use was included (no/yes). Those endorsing this question were asked about their experiences thereof. Due to the lack of standardized scales about this topic, ten items were constructed for the purpose of the present study reflecting the following experiences: (1) made me prioritize sleep more, (2) helped me sleep better, (3) made me more curious about my sleep, (4) I trust results from the sleep app more than my subjective experience, (5) the sleep app results correspond with my personal experience, (6) the sleep app tells the truth about my sleep, (7) the use of sleep app has been positive, (8) the sleep app made me more concerned about my sleep, (9) the sleep app made me more stressed about my sleep, and (10) the sleep app made me more conscious about my sleep. The response alternatives for the ten items were aligned with a 5-point Likert scale ranging from 1 (totally disagree) to 5 (totally agree).

### 2.3 Statistical analysis

Data were analyzed with IBM SPSS, version 29.0. Descriptive analyses comprised estimation of means, standard deviations and proportions. In order to examine characteristics associated with use of sleep apps (no/yes) a logistic regression analysis was performed, where age, sex, education, marital status, childcare responsibility, morningness, insomnia and the HEXACO-personality model (Honesty-humility, Emotionality, Extraversion, Agreeableness, Conscientiousness, Openness to experience) constituted the independent variables. A factor analysis (maximum likelihood) was conducted for the 10 experienced effects of sleep app use, and factors with an Eigenvalue ≥1.00 were retained. The Kaiser-Meyer-Olkin measure of sampling adequacy was 0.80 and the Bartlett's test of sphericity was significant (*p* < 0.001), attesting to the factorability of the data (Tabachnick and Fidell, 2013). To address the possibility of multiple factors being retained, direct oblim rotation was utilized, and a composite score was created for each factor, including items loading at least 0.60 on the factor (Osborne, 2014). Linear multiple regressions were then performed, where the composite scores of the items loading on the factors in question, constituted the dependent variables, and the independent variables were the same as for the logistic regression analysis. Preliminary analyses were conducted to ensure no violation of the assumption of normality, linearity, multicollinearity and homoscedasticity.

## 3 Results

### 3.1 Use of sleep apps

A total of 346 respondents (36.8%) reported using or having used a sleep application. For the logistic regression analysis, data for the 13 respondents who identified themselves as “other” in terms of gender were removed as the number of respondents in this group was deemed insufficient for meaningful inclusion in the analyses. The results are shown in Table 1. Age (OR = 1.07, 95% CI = 1.02–1.12), insomnia symptoms (OR = 1.02, 95% CI = 1.01–1.04) and conscientiousness (OR = 1.04, 95% CI = 1.01–1.07) were positively and significantly related to sleep app use. Table 2 depicts the distribution of the responses to the items assessing experiences with sleep app use. Most of the responses seemed to center on the three middle (i.e., disagree, neither agree nor disagree, and agree) alternatives.

**Table 1:** Logistic regression predicting use of sleep app (No = 0, Yes = 1; *N* = 927).

**Variable**	**OR**	**95% CI**
Age	**1.07**	**1.02** ***–*** **1.12**
Sex (♂ = 1, ♀ = 2)	1.04	0.75–1.44
**Education**		
Low^a^	0.82	0.59–1.15
High^a^	0.97	0.68–1.40
Marital status (single = 1, married/partner = 2)	1.01	0.75–1.37
Children (1 = no, 2 = yes)	0.81	0.51–1.27
MEQ-reduced	1.04	1.00–1.08
Bergen Insomnia scale	**1.02**	**1.01** ***–*** **1.04**
Honesty-humility	0.99	0.97–1.01
Emotionality	1.00	0.98–1.02
Extraversion	1.00	0.98–1.02
Agreeableness	1.01	0.98–1.03
Conscientiousness	**1.04**	**1.01** ***–*** **1.07**
Openness to experience	1.00	0.98–1.02

a Middle education is contrast. The model was significant (χ^2^ = 33.9, df = 14, p = 0.002), Nagelkerke R^2^ = 0.049. Significant associations are shown in bold.

**Table 2:** Distribution of responses to the items reflecting experiences with sleep app use (*N* = 346).

	**Totally disagree**	**Disagree**	**Neither agree nor disagree**	**Agree**	**Totally agree**
Using the sleep app made me prioritize sleep more	13.0%	29.2%	18.8%	34.1%	4.9%
Using the sleep app helped me sleep better	16.2%	38.4%	26.3%	15.3%	3.8%
Using the sleep app made be more curious about my sleep	2.6%	3.8%	10.1%	56.9%	26.6%
I trust the results from the sleep app more than my subjective experience of my sleep	7.8%	28.9%	30.6%	26.6%	6.1%
The results from the sleep app correspond well with my personal experience of my sleep	3.8%	17.6%	26.6%	48.0%	4.0%
The results from the sleep app tell the truth about my sleep	4.6%	15.9%	34.7%	41.6%	3.2%
Overall using the sleep app has been positive for me	4.6%	16.2%	33.8%	39.9%	5.5%
Using the sleep app made me more concerned about my sleep	7.5%	15.6%	12.4%	54.3%	10.1%
Using the sleep app made be more stressed about my sleep	11.0%	33.8%	17.6%	32.4%	5.2%
Using the sleep app made be more conscious about my sleep	4.0%	6.9%	7.8%	65.0%	16.2%

### 3.2 Factor analyses

A total of three factors were retained (Eigenvalues > 1.00). The factor loadings are shown in Table 3. Factor 1 reflected three items and was named “trust/objectification”, as the items reflected a strong belief in the validity of the sleep app measurements. Two items loaded on the second factor, named “negative effects,” and two items loaded on the third factor, named “positive effects”.

**Table 3:** Pattern Matrix of The Factor Analysis Following Oblim Rotation (*N* = 346).

	**Factor 1**	**Factor 2**	**Factor 3**
	**Trust/objectification**	**Negative effects**	**Positive effects**
Using the sleep app made me prioritize sleep more	−0.051	0.133	−**0.837**
Using the sleep app helped me sleep better	−0.025	−0.041	−**0.875**
Using the sleep app made be more curious about my sleep	0.415	0.215	−0.044
I trust the results from the sleep app more than my subjective experience of my sleep	**0.601**	0.100	−0.027
The results from the sleep app correspond well with my personal experience of my sleep	**0.879**	−0.080	0.049
The results from the sleep app tell the truth about my sleep	**0.839**	−0.045	0.050
Overall using the sleep app has been positive for me	0.536	−0.264	−0.443
Using the sleep app made me more concerned about my sleep	0.132	**0.682**	−0.207
Using the sleep app made be more stressed about my sleep	−0.119	**0.782**	0.099
Using the sleep app made be more conscious about my sleep	0.232	0.484	−0.155

Loadings ≥0.60 are shown in bold.

### 3.3 Linear regression analyses

The regression model (Table 4) for trust/objectification was significant [*F*_(14, 329)_ = 2.61, *p* < 0.01, *R*^2^_adj_ = 0.062]. Age (β = 0.149, *p* = 0.010) and agreeableness (β = 0.112, *p* = 0.045) were positively related to trust/objectification, whereas high education (β = −0.116, *p* = 0.043) and openness to experience (β = –0.163, *p* = 0.003) both were inversely related to trust/objectification. Table 5 shows the regression model of negative effects of sleep app use. Overall, the model was not significant [*F*_(14, 329)_ = 1.59, *p* > 0.05, *R*^2^_adj_ = 0.021]. Still two independent variables, insomnia (β = 0.185, *p* = 0.002) and emotionality (β = 0.154, *p* = 0.017), were positively associated with negative effects. The regression model for positive effects of sleep app use was significant [*F*_(14, 329)_ = 1.83, *p* < 0.05, $R_{\text{adj}}^{2}$ = 0.033]. The results are shown in Table 6. Morningness was positively (β = 0.141, *p* = 0.015) related to the positive effects of sleep app use, whereas gender (♂ = 1, ♀ = 2; β = –0.131, *p* = 0.038) and high education (β = –0.172, *p* = 0.002) were inversely related.

**Table 4:** Regression analysis summary for demographic, sleep related and personality variables predicting trust/objectification (*N* = 344).

**Variable**	***B***	**SE *B***	**β**	***t***	***p***
Age	0.119	0.046	0.149	2.584	0.010
Sex (♂ = 1, ♀ = 2)	−0.171	0.303	−0.035	−0.563	0.574
Education					
Low^a^	0.158	0.319	0.028	0.496	0.620
High^a^	−0.693	0.341	−0.116	−2.030	0.043
Marital status (single = 1, arried/partner = 2)	0.018	0.296	0.004	0.062	0.951
Children (1 = no, 2 = yes)	−0.081	0.423	−0.011	−0.191	0.849
MEQ-reduced	0.029	0.036	0.047	0.817	0.415
Bergen Insomnia scale	−0.019	0.016	−0.073	−1.238	0.217
Honesty-humility	−0.015	0.023	−0.038	−0.660	0.510
Emotionality	0.024	0.022	0.071	1.223	0.262
Extraversion	0.037	0.020	0.109	1.875	0.062
Agreeableness	0.047	0.023	0.112	2.014	0.045
Conscientiousness	−0.026	0.023	−0.061	−1.062	0.289
Openness to experience	−0.056	0.019	−0.163	−2.981	0.003

a Technical or community college/undergraduate degree comprised the reference category.

**Table 5:** Regression analysis summary for demographic, sleep related and personality variables predicting negative effects (*N* = 344).

**Variable**	***B***	**SE *B***	**β**	***t***	***p***
Age	0.040	0.037	0.063	1.067	0.287
Sex (♂ = 1, ♀ = 2)	−0.121	0.246	−0.031	−0.489	0.625
Education					
Low^a^	0.011	0.259	0.002	0.041	0.967
High^a^	0.110	0.277	0.023	0.397	0.692
Marital status (single = 1, arried/partner = 2)	−0.093	0.232	−0.024	−0.399	0.690
Children (1 = no, 2 = yes)	−0.359	0.343	−0.062	−1.046	0.296
MEQ-reduced	0.034	0.029	0.068	1.177	0.240
bergen insomnia scale	0.039	0.013	0.185	3.050	0.002
Honesty-humility	0.014	0.018	0.047	0.793	0.429
Emotionality	0.042	0.018	0.154	2.393	0.017
Extraversion	0.014	0.016	0.050	0.845	0.399
Agreeableness	−0.007	0.019	−0.020	−0.345	0.730
Conscientiousness	0.012	0.019	0.036	0.613	0.540
Openness to experience	−0.014	0.015	−0.051	−0.919	0.395

a Technical or community college/undergraduate degree comprised the reference category.

**Table 6:** Regression analysis summary for demographic, sleep related and personality variables predicting positive effects (*N* = 344).

**Variable**	***B***	**SE *B***	**β**	***t***	***p***
Age	0.036	0.039	0.054	0.924	0.356
Sex (♂ = 1, ♀ = 2)	−0.537	0.257	−0.131	−2.088	0.038
Education					
Low^a^	−0.366	0.270	−0.078	−1.354	0.177
High^a^	−0.860	0.289	−0.172	−2.972	0.003
Marital status (single = 1, arried/partner = 2)	0.247	0.243	0.060	1.017	0.310
Children (1 = no, 2 = yes)	0.211	0.358	0.035	0.590	0.555
MEQ-reduced	0.074	0.030	0.141	2.442	0.015
Bergen Insomnia scale	0.015	0.013	0.067	1.114	0.266
Honesty-humility	−0.016	0.019	−0.049	−0.826	0.409
Emotionality	0.021	0.018	0.075	1.165	0.245
Extraversion	0.006	0.017	0.020	0.343	0.732
Agreeableness	0.020	0.020	0.057	1.002	0.317
Conscientiousness	−0.026	0.020	−0.078	−1.339	0.182
Openness to experience	0.023	0.016	0.079	1.427	0.155

a Technical or community college/undergraduate degree comprised the reference category.

## 4 Discussion

The study showed that a considerable proportion, about 37%, of the sample used or had used sleep apps. A previous study of primary care patients revealed that about 48% employed at least one smartphone health app, with usage being more prevalent among those below 30 years of age (Paradis et al., 2022). A national study among US mobile phone owners showed that 58% had downloaded a health-related mobile app (Krebs and Duncan, 2015). However, the proportion of the participants in the two aforementioned studies who specifically used a sleep app is not known. As such the current study is one of the first that has estimated the proportion of sleep app users specifically.

Within the current sample with age range 18–30 years, age was positively associated with sleep app use, probably due to an increased likelihood of encountering such apps with increasing age. Previous studies on nationally representative samples have shown that younger individuals are generally more likely to use health apps compared to older adults (Carroll et al., 2017). The current study thus indicates that the relationship between app use and age may depend on the age range of the sample.

Insomnia symptoms were positively associated with sleep app use. This aligns with studies showing that people with insomnia have sleep-related attentional bias (Harris et al., 2015). Hence, people with insomnia can be expected to be more likely to notice and start using sleep apps than others. Further, it seems reasonable that individuals experiencing sleep difficulties are more likely to seek out information and potential aids (including apps) to address the issues they face. Indeed, the link between insomnia symptoms and sleep app use is also in line with studies showing that people suffering from insomnia to a higher degree than people without insomnia resort to various tricks and strategies to help them sleep (Bjorvatn et al., 2023; Ree and Harvey, 2004). Furthermore, we found that conscientiousness was positively associated with sleep app use. Although conscientiousness in general has been found to be inversely related to mobile app use, the relationship appears to be contingent upon the type of mobile app, and in one study, conscientiousness was found to be positively related to health app use, most likely reflecting the fact that people high on conscientiousness also tend to score higher on positive health behaviors (Burtaverde et al., 2021).

The following discussion pertains to the three factors reflecting the user experiences. Trust/objectification was positively associated with age. This may reflect that younger people are more critical consumers of apps than older people. Still is should be noted that the age range in the present study was rather limited. Our findings further revealed that agreeableness was positively associated with trust/objectification. This may be related to the innate tendency of people high on agreeableness to seek harmony (Graziano and Tobin, 2009) and to avoid conflicts (Tehrani and Yamini, 2020), hence not contesting the sleep app results. People with higher levels of education were found to have lower scores on the trust/objectification factor compared to those with middle levels of education. This is partly in line with studies showing that people with higher education seek more information about products they purchase compared to their lower educated counterparts (Besler et al., 2012), which conceivably prone them to be more censorious users. The present study further found that openness to experience was inversely related to trust/objectification. Openness to experience reflects the ability to learn, and capacity for knowledge and understanding (McCrae and Costa, 1997), which presumably make them more critical consumers. People suffering from insomnia are known to be prone to worry (Pallesen et al., 2002) and to be reactive to both previous and upcoming events (Kalmbach et al., 2016) and elevated reactivity is a hallmark of people high on emotionality (Bolger and Schilling, 1991). These associations may explain the finding that negative effects were positively related to both insomnia and emotionality. Further, individuals with insomnia are known to focus excessively on their sleep, which plays a key role in perpetuating insomnia (Harvey, 2002). While sleep app use may be well-intentioned, increasing focus on sleep without proper evidence-based guidance may lead to frustration or worsen the sleep problem for those with insomnia. The results of the present study could potentially reflect such unintended consequence for those who struggle with their sleep, where heightened attention on sleep, rather than resolving their problems, uphelds the viscous cycle of insomnia (Fabbri et al., 2023). Males scored higher than females on the positive effects dimension, which is difficult to explain as males have been shown to prefer fitness applications, whereas females report a higher preference for applications related to nutrition, self-healthcare, and reproduction (Wang and Qi, 2021). Still, the finding may reflect that men more often than women are technologically adept (Goswami and Dutta, 2016). Morningness was positively associated with positive effects and might reflect assumingly more positive feedbacks being received from sleep apps among morning larks compared to night owls, as the former is generally known to sleep better than the latter (Minz and Pati, 2021). Those with high levels of education reported lower positive effects of sleep apps than those with middle levels of education, which may, as for the negative effects, be explained by higher level of product seeking information in the former group (Besler et al., 2012).

## 5 Strengths and limitations

The present study is one of the very first that seek to characterize users of sleep apps and their experiences thereof. Another asset of the current study is the identification of various dimensions of experiences, which resulted in three factors. Still, these dimensions should be regarded as tentative, and more stringent approaches to scale developments (Boateng et al., 2018; Carpenter, 2018) in this realm are recommended for future research. In this regard it should be noted that the items assessing sleep app use experiences were constructed based on reviews of the current and limited literature on the topic as well as relevant discourses (positive vs. negative effects and degree of correspondence with polysomnography) about sleep apps (Al Mahmud et al., 2022; Van den Bulck, 2015). Still, there is some risk that significant other impacts of experiences were not captured by the included items. The use of validated instruments regarding the independent variable ensured proper assessment of the majority of constructs under investigation and the sample was of reasonable size. In addition, steps were taken to remove data from respondents with careless responding. Still, some limitations should be mentioned. The sample was a convenience sample, which restricts the generalizability of the findings. Using Prolific to recruit participants also presents other potential challenges for generalizability. Those who participate on such platforms often consist of individuals who frequently engage in online surveys or experiments, which may create a bias toward more tech-savvy or higher-educated individuals. Additionally, while the platform aims to provide diverse samples, there is concern that certain demographic groups may be overrepresented (e.g., younger adults). Also, since participants are paid for their time, some may prioritize maximizing earnings over fully engaging with the study, which can compromise data quality. Moreover, the regular involvement of certain participants in studies can lead to the emergence of “professional participants,” introducing further bias into the research results. Still, compared to other online survey platforms Prolific seems to provide a high proportion of high quality respondents (Douglas et al., 2023). A cross-sectional design was used, which hampers conclusions about directionality and causality. This may also introduce biases such as the common method bias (Podsakoff et al., 2003). Future studies should therefore emphasize longitudinal and experimental designs. Some of the independent variables, such as the personality dimensions, where, despite some overall associations with sleep (Duggan and Križan, 2019), included mainly due to explorative purposes and reflect as such the overall limited amount of empirical and theoretical publications in the field. The explained variance in the regression models was low, indicating that additional independent variables may be relevant and should be incorporated in future studies. Furthermore, the study did not differentiate between amount of use, type of platform used, and type of sleep app functions used in terms of the experienced effects. Hence, this clearly warrants a more detailed approach in future research. Additionally, future studies should explore the reasons for why people start as well as stop using sleep apps. It should also be noted that sleep app use was exclusively assessed through self-report, hence future studies should corroborate such reports against objective recordings of use. The age range of the sample was quite confined, hence the findings should be corroborated in samples with a wider age range.

## 6 Conclusions

In a sample of young adults, we found that about one third used or had used sleep apps. Sleep app use was positively associated with age, insomnia symptoms and conscientiousness. Three dimensions of experiences with sleep apps were identified, reflecting high trust (trust/objectification) in sleep apps, as well as perceived negative and positive effects. People with insomnia and high scores on emotionality reported more negative effects than their counterparts. Males and morning types reported more positive effects than females and evening types, whereas less positive effects were reported by those with high education. These findings indicate that individual characteristics play a role in determining the perceived effects of sleep apps, and both sleep app manufacturers and practitioners should bear this in mind when recommending sleep apps to clients and others.

## Data Availability

The raw data supporting the conclusions of this article will be made available by the authors, without undue reservation.
